# Ti_3_C_2_T_x_ MXene Quantum Dots with Surface-Terminated Groups (-F, -OH, =O, -Cl) for Ultrafast Photonics

**DOI:** 10.3390/nano12122043

**Published:** 2022-06-14

**Authors:** Jianfeng Liu, Shanshan Chen, Junshan He, Runming Huang, Lili Tao, Yu Zhao, Yibin Yang

**Affiliations:** 1 School of Materials and Energy, Guangdong University of Technology, Guangzhou 510006, China; 2112002059@mail2.gdut.edu.cn (J.L.); hjs3108007371@163.com (J.H.); 3120006078@mail2.gdut.edu.cn (R.H.); taoll@gdut.edu.cn (L.T.); zhaoyu@gdut.edu.cn (Y.Z.); yangyibin@gdut.edu.cn (Y.Y.); 2 Guangdong Provincial Key Laboratory of Information Photonics Technology, Guangdong University of Technology, Guangzhou 510006, China

**Keywords:** Ti_3_C_2_T_x_ quantum dots, surface functional groups, tapered fiber saturable absorber, mode-locked, Er^3+^-doped fiber laser

## Abstract

Transition metal carbides and nitrides (MXenes) have attracted significant attention in photoelectric applications due to their highly tunable electronic and optical properties influenced by a flexible compositional or surface functional group regulation. Ti_3_C_2_T_x_ MXenes (-F, -OH, =O terminated) used in previous ultrafast photonic studies are usually synthesized via a generic hydrofluoric acid (HF) etching strategy, which may cause numerous defects and thus impedes the optoelectronic properties of Ti_3_C_2_T_x_. In this contribution, inspired by a much higher conductivity and carrier mobility of Ti_3_C_2_T_x_ (-F, -OH, =O, -Cl terminated) prepared from a minimally intensive layer delamination method (MILD) etching strategy, we further optimized it with a liquid-phase exfoliation (LPE) method to synthesize pure Ti_3_C_2_T_x_ quantum dots (QDs) for ultrafast photonic. Compared to the other QDs saturable absorber (SA) devices performed at 1550 nm, our SA device exhibited a relatively low saturation intensity (1.983 GW/cm^−2^) and high modulation depth (11.6%), allowing for a more easily mode-locked pulse generation. A distinguished ultrashort pulse duration of 466 fs centered at the wavelength of 1566.57 nm with a fundamental frequency of 22.78 MHz was obtained in the communication band. Considering the SA based on such a Ti_3_C_2_T_x_ QDs tapered fiber is the first exploration of Er^3+^-doped fiber laser (EDFL), this work will open up a new avenue for applications in ultrafast photonics.

## 1. Introduction

An ultrafast laser which shows short pulse duration from picosecond to femtosecond regions and high pulse energy has garnered tremendous attention due to the increasing demands and widespread applications in industrial production and scientific research [1]. Passive mode-locking operation, which takes advantage of a simpler saturable absorber (SA) as optical loss modulator, shows the merits of a fast recovery time, low cost, and compactness s [2,3]. To some extent, passive operation with flexibility is more in line with the direction of industrial development. Materials with nonlinear optical properties are commonly used as an SA, which is one of the most important parts of an ultrafast laser system [4,5]. Currently, semiconductor saturable absorber mirrors (SESAMs) are widely developed and used as commercial SAs. Although SESAMs exhibit outstanding performance in stability, they still face restrictions due to their complex fabrication process, expensive preparation cost, and narrow working wavelength [6,7]. Therefore, more and more efforts have been paid to explore new suitable SA materials.

A lot of low-dimensional materials [8,9], including two-dimensional (2D), one-dimensional (1D), and zero-dimensional (0D) materials, show relatively excellent nonlinear optical properties. In recent years, these low-dimensional materials have been utilized for ultrafast pulse laser generation in broad wavelength regions. Graphene [10] was the first discovered 2D material as well as the earliest SA material. Its fast carrier recovery time and broadband saturable absorption characteristics opened the door to ultrafast pulse laser applications for 2D materials. Unfortunately, its low absorption coefficient and low damage threshold constrained graphene from developing in this field [11]. Therefore, many researchers began to shift focus on other low-dimensional materials, such as single-walled carbon nanotubes (SWCNTs) [12], black phosphorus (BP) [13,14], transition metal dichalcogenides (TMDs) [15,16,17], topological insulators (TIs) [18,19], perovskite [20,21], metal–organic frameworks (MOFs) [22,23,24], transition metal carbides and nitrides (MXenes) [25,26,27], and various quantum dots (QDs) [28,29,30,31]. However, there are still many bottlenecks, such as nontunable bandgap, unstable structures, or unstable optical modulation performance. Further research on material preparation and ultrafast photonics applications are still urgently needed.

Among these materials, MXenes have attracted huge attention due to their outstanding photoelectric characteristics and adjustable performance caused by flexible compositional or surface functional group regulation [32,33]. Ti_3_C_2_T_x_ (T_x_ represents surface terminated groups) is the first synthetic and the most popular MXenes [34]. Its nonlinear optical absorption properties have been investigated and mode-locking femtosecond pulses have been successfully generated by using Ti_3_C_2_T_x_ as an SA [27]. Similar to graphene, Ti_3_C_2_T_x_ is metallic and exhibits broadband applicability for pulse lasers, even down to the 3000 nm midinfrared range [35]. In addition to these versatile photonic applications based on Ti_3_C_2_T_x_ films, 0D Ti_3_C_2_T_x_ QDs still show a wideband nonlinear optical response from 540 nm to 1550 nm [28], and they have been used to generate ultrashort pulses in an erbium- and ytterbium-doped fiber laser cavity. It is worth noting that QDs have gained a marked interest for their promising prospects in nonlinear optics during the past two years. The enhanced nonlinear effects caused by quantum confinement effects [36,37] promote the development of QDs SA. It has been observed that ultrafast pulses were generated relatively easily for Ti_3_C_2_T_x_ QDs compared with Ti_3_C_2_T_x_ nanosheets (NSs) [27,29]. However, reports about ultrafast optical devices based on Ti_3_C_2_T_x_ QDs have been very limited to date [27,29], which calls for further exploration and supplementary research.

Ti_3_C_2_T_x_ MXenes (-F, -OH, =O terminated) used in previous ultrafast photonic studies were based on a generic hydrofluoric acid (HF) etching strategy [26,27,28]. However, HF is highly corrosive, and the etching process results in issues related to toxicity. More importantly, severe etching reactions can also cause the formation of numerous surface defects, which serious impedes the optoelectronic properties. Recently, fluoride-based salt etching methods have been gradually optimized. Among them, a method to generate in situ HF by combining lithium fluoride (LiF) and hydrochloric acid (HCl), the so called minimally intensive layer delamination (MILD) strategy [38], has been widely promoted in various studies. Encouragingly, the Ti_3_C_2_T_x_ MXenes produced by this strategy commonly terminated extra Cl groups, which have a better crystal quality and a much higher conductivity and carrier mobility than ones prepared by HF [39]. Furthermore, a more recent study [40,41] based on Ti_3_C_2_Cl also suggested that Cl groups facilitate electrons transfer. The above studies imply that 0D Ti_3_C_2_T_x_ MXenes produced by the MILD strategy is more suitable for ultrafast photonics research.

Inspired by the aforementioned studies, we exploited a MILD combined with a liquid-phase exfoliation (LPE) method to obtain Ti_3_C_2_T_x_ QDs with surface-terminated groups (-F, -OH, =O, -Cl) for ultrafast photonics. Since the long-term stability of low-dimensional MXenes still faces challenges and the material properties are closely correlated to the terminated surface groups, the structures, characteristics of morphology, and compositions were analyzed in detail and reported in the following paragraphs. Then, a saturable absorber device was fabricated by a photodeposition method and the nonlinear response of the Ti_3_C_2_T_x_ QDs tapered fiber SA was measured at a central wavelength of 1550 nm. The modulation depth and saturation intensity were estimated to be 11.6% and 1.983 GW/cm^−2^, respectively. Finally, an ultrafast and ultranarrow pulse with pulse duration of 466 fs and fundamental frequency of 22.78 MHz was operated in the Er^3+^-doped fiber laser (EDFL) cavity based on the tapered fiber SA. The results indicated that the Ti_3_C_2_T_x_ QDs (-F, -OH, =O, -Cl terminated) tapered fiber SA shows excellent potential application in ultrafast laser devices. It provides a new approach for the application of Ti_3_C_2_T_x_ QDs in ultrafast photonics.

## 2. Materials and Methods

### 2.1. Preparation of Ti_3_C_2_T_x_ QDs

**Figure 1:**
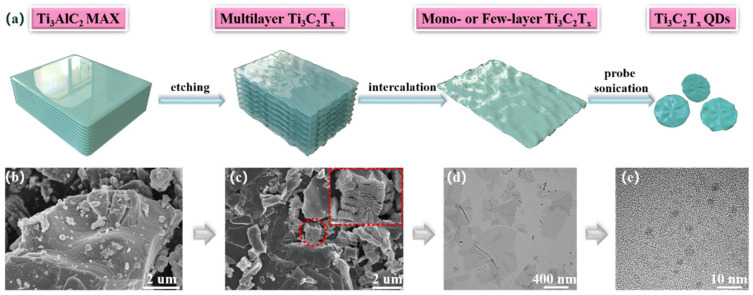
(**a**) Schematic diagram for the preparation of Ti_3_C_2_T_x_ QDs. (**b**) SEM image of Ti_3_AlC_2_ MAX. (**c**) SEM image of multilayer Ti_3_C_2_T_x_ NSs. (**d**) TEM image of mono- or few-layer Ti_3_C_2_T_x_ NSs. (**e**) TEM image of Ti_3_C_2_T_x_ QDs.

The preparation process of Ti_3_C_2_T_x_ QDs is shown in Figure 1a. Ti_3_C_2_T_x_ QDs were obtained from the precursor of Ti_3_AlC_2_ MAX phase (the layered carbide/nitride-derived phases, where M is an early transition metal, X is N or C, and A is typically a group 13 or 14 element, e.g., aluminum, MXenes are obtained by etching A element away from the MAX phase) through a series of treatments, such as etching, intercalation, and ultrasonic exfoliation. The specific experimental steps were as follows:

The first step was etching the MAX phase to synthesize Ti_3_C_2_T_x_ NSs. The method used to gain Ti_3_C_2_T_x_ NSs was similar to the MILD method [38]. It was further optimized with an LPE method through a high-power ultrasonic cell smash in this work. The mixture of 3.2 g lithium fluoride (99.99%, Macklin, Shanghai, China) and 40 mL 9 M hydrochloric acid (36 wt %, Guangzhou, Guangzhou, China) was served as the etchant. Firstly, it was stirred in a 100 mL polytetrafluoroethylene beaker for 30 min to achieve complete dissolution. Then, 2.0 g Ti_3_AlC_2_ (400 mesh, 11 Technology, Jilin, China) was slowly added to the etching solvent. The speed of the stirrer was set to 400 rpm, and the etching reaction was set at 35 °C for 24 h. It is worth noting that there should be a small gap at the mouth of the beaker to release the gas produced during the reaction. The entire procedure was carried out in a fume hood. To remove the excess LiF and coproduct AlF_3_, 1 M HCl was used to wash the reaction product by centrifugation (H1850, Cence, Hunan, China) 2–3 times after the reaction. A subsequent wash with deionized water was carried out 5–7 times until the pH of the supernatant reached 6, and then the solution of multilayer Ti_3_C_2_T_x_ was obtained. The next step was a better delamination with an ice bath ultrasonication (KQ-500VDE, ShuMei, Jiangsu, China) for 2 h until the sediment swelled. The mono- or few-layer Ti_3_C_2_T_x_ in dark green supernatant was collected by repeatedly centrifuging at 3500 rpm for 1 h. A freezing dryer (LC-12N-50A, LiChen, Shanghai, China) was used to ensure that the mono- or few-layer Ti_3_C_2_T_x_ was unoxidized during the dehydration process. A portion of the fully dried mono- or few-layer Ti_3_C_2_T_x_ was used for characterization, and the remaining portion was used to prepare quantum dots by probe sonication (Scientz-II, SCIENTZ, Zhejiang, China) in ethanol solution (99.7%, Sun, Zhiyuan, Tianjin, China) for 12 h. Finally, the Ti_3_C_2_T_x_ QDs were collected by centrifugation twice at 10,000 rpm for 1 h and distilled to 10 mL.

### 2.2. Preparation of SA Device Based on Ti_3_C_2_T_x_ QDs

The single-mode fiber (SMF-28e, Nufern, East Granby, CO, USA) was stretched into a tapered fiber with a conical length of 9.36 mm and a width of 10 μm by using a fiber TEC machine (XQ71C0, Oscom Technology, Shenzhen, China). Then, the photodeposition method [42] was employed to stably transfer Ti_3_C_2_T_x_ QDs to the tapered area to fabricate the SA. A specific schematic diagram of the SA device is exhibited in Figure S1.

### 2.3. Characterization Methods

The surface morphology was observed by field emission scanning electron microscopy (SEM, SU8010, HITACHI, Tokyo, Japan). The further morphology, crystalline structure, and elemental profiles were investigated by transmission electron microscope (TEM, F200S, Talos, Thermo Fisher Scientific, Waltham, MA, USA) equipped with an energy-dispersive X-ray spectroscope (EDS). The phase structure was identified by an X-ray diffractometer (XRD, D8 Advance, Bruker, Billerica, MA, USA). The thickness and particle size were measured using a scanning probe microscope (SPM, Dimension FastScan, Bruker, Billerica, MA, USA). The Raman spectra were obtained using a Raman spectrometer (FEX, NOST, Seongnam-si, Korea) with a 532 nm argon-ion excitation laser. The absorption spectrum was acquired by using a UV–vis–NIR spectrophotometer (UV-3600 Plus, SHIMADZU, Tokyo, Japan).

## 3. Results

### 3.1. Characterization of Ti_3_C_2_T_x_

The morphology images corresponding to the preparation diagram are shown in Figure 1b–e. Figure 1b, c both are SEM images, which demonstrate the formation of an accordion-like structured Ti_3_C_2_T_x_ MXene by etching the Ti_3_AlC_2_ MAX phase with tight interlayer bonds. Figure 1d is a TEM image of mono- or few-layer Ti_3_C_2_T_x_ NSs through in situ Li^+^ ions intercalation, which presents a unique wrinkled morphology of an ultrathin two-dimensional nanostructure. Subsequently, the NSs were exploited to prepare Ti_3_C_2_T_x_ quantum dots (QDs), as seen in Figure 1e.

**Figure 2:**
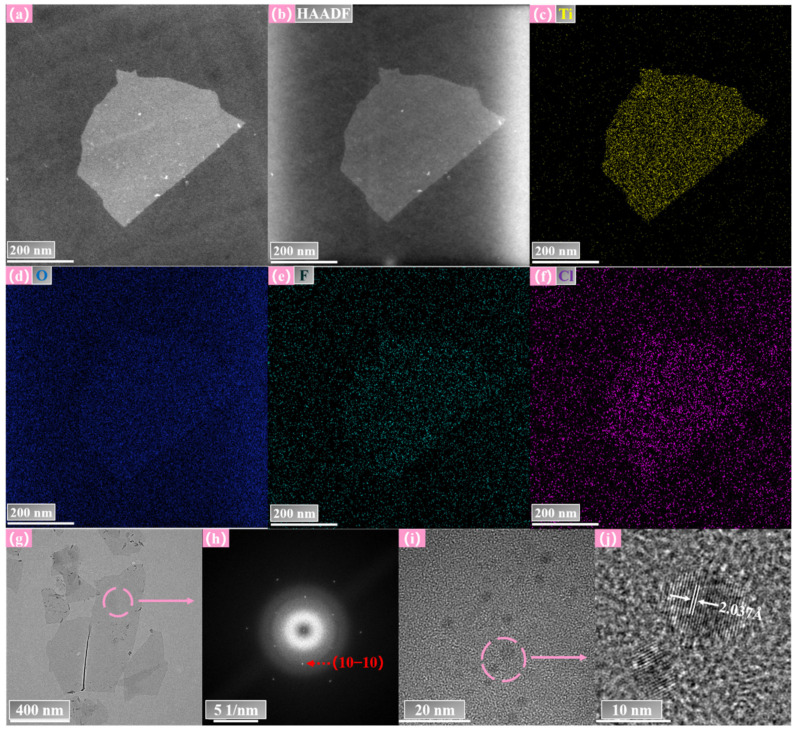
(**a**) TEM image of Ti_3_C_2_T_x_ NS. (**b**) HAADF image of the Ti_3_C_2_T_x_ NS. (**c**–**f**) EDS elemental mappings of Ti, O, F, and Cl, respectively, for the Ti_3_C_2_T_x_ NS. (**g**) Ultrathin Ti_3_C_2_T_x_ NSs with distinctive wrinkled morphology. (**h**) The SEAD diagram corresponds to the pink area in (**g**). (**i**) TEM image of Ti_3_C_2_T_x_ QDs. (**j**) Quantum dots with distinct lattice stripes correspond to the pink region in (**i**).

The TEM images of as-prepared Ti_3_C_2_T_x_ NSs confirmed that all these flakes displayed typical 2D morphological characteristics as shown in Figure 2a,g. The high-angle annular dark-field (HAADF) image and EDS mappings for Ti, O, F, and Cl elements corresponding to the NS in Figure 2a are exhibited in Figure 2b–f. The signal of Ti was strong, and signals of O, F, and Cl were relatively weak. These elements were evenly distributed. It indicated that F and Cl from the reactants were uniformly doped in the NS. O corresponds to the adsorbed oxygen or common -OH terminal groups. The signal of the main element C was masked by the carbon matrix. Different from the MXenes always terminated by O, OH, and F via the traditional HF etching method, the products showed the terminal groups of a combination of Cl and the above species. Since surface functional groups directly determine the photoelectric properties of Ti_3_C_2_T_x_, the extra Cl groups are likely to exhibit special optical properties. The selected area electron diffraction (SAED) patterns of the NS corresponding to Figure 2g show that the Ti_3_C_2_T_x_ NS possesses a hexagonal structure (as shown in Figure 2h). The diffraction spots near the center correspond to the $\left\{10\bar{1}0\right\}$ crystal planes of Ti_3_C_2_T_x_. According to the high-magnification TEM images, the as-synthesized Ti_3_C_2_T_x_ QDs dispersed in ethanol presented a uniform size and distribution with distinct boundaries (as shown in Figure 2i,j). The QDs were spherical with diameters of 5–10 nm. As shown in Figure 2j, the lattice fringes had an inner plane spacing of 2.037 Å, which related to the $\left(10\bar{1}0\right)$ facet of the Ti_3_C_2_T_x_. The clear diffraction spots and lattice fringes verified that both the Ti_3_C_2_T_x_ NSs and QDs had good crystal quality.

**Figure 3:**
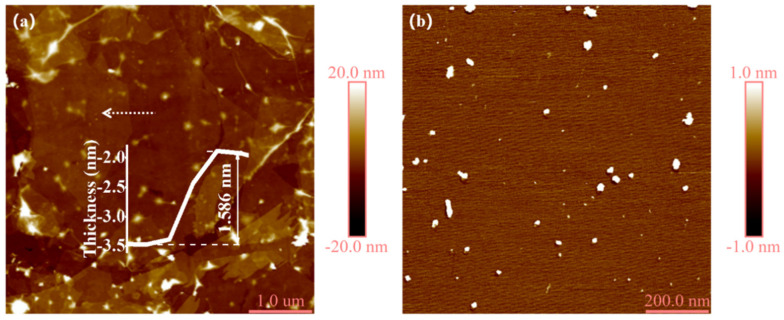
SPM images of Ti_3_C_2_T_x_ NSs (**a**) and Ti_3_C_2_T_x_ QDs (**b**), respectively.

In addition, the thickness of the Ti_3_C_2_T_x_ NSs was investigated by SPM. Here, a Ti_3_C_2_T_x_ film obtained by self-assembly [43] of the NSs was used, because the NSs needed to be dispersed in a solvent and the signal from the solvent interfered with the results (details about the self-assembly method are explained in Figure S2 of the supplementary information). As shown in Figure 3a, the area was composed of multiple overlapping NSs. The thickness of the NS was 1.586 nm, which is consistent with the thickness of a single-layer Ti_3_C_2_T_x_ NS as reported in the literature. It is noted that the NSs were so thin that there were many folds in the assembled film, which is the bright area in Figure 3a. Meanwhile, the size and distribution of the Ti_3_C_2_T_x_ QDs were investigated as shown in Figure 3b. It revealed the Ti_3_C_2_T_x_ MXene QDs were uniform and ultrasmall. The SPM analysis (Figure S3) demonstrated that the thickness of the QDs was around 1.8 nm and the average diameter was around 17.7 nm via statistical analysis of 50 MXene QDs. The average lateral size was slightly larger than the TEM result, which may be caused by a low longitudinal resolution because of the low scanning frame number during SPM testing.

**Figure 4:**
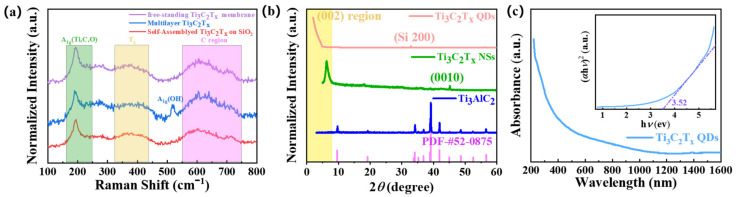
(**a**) Raman spectra of Ti_3_C_2_T_x_ NSs with various states. (**b**) XRD patterns of Ti_3_AlC_2_ MAX, Ti_3_C_2_T_x_ NSs, and Ti_3_C_2_T_x_ QDs deposited on a silicon wafer, respectively. (**c**) UV–vis–NIR absorption spectrum of Ti_3_C_2_T_x_ QDs; the inset is the value of the bandgap fitted by using a Tauc method.

Raman spectra and XRD patterns were carried out to further determine the structural information of the material. The typical Raman spectrum of Ti_3_C_2_T_x_ commonly has multiple features in the 100–800 cm^−1^ range. In this study, the Raman spectra of various MXene materials containing self-assembled Ti_3_C_2_T_x_ film on a quartz substrate, the free-standing Ti_3_C_2_T_x_ membrane obtained by the extraction method, multilayer Ti_3_C_2_T_x_ NSs, and Ti_3_C_2_T_x_ QDs on silicon substrate are presented in Figure 4a,b. The Raman spectrum of the precursor MAX phase was similar to other report, as shown in Figure S4a (details can be found in the supplementary information). Moreover, the presented spectra of the Ti_3_C_2_T_x_ samples were noticeably different from that of the MAX phase. Firstly, an obvious sharp peak at 197 cm^−1^ corresponded to the A_1g_ (Ti, C, O) mode, which was an out-of-plane vibration of Ti atoms in the outer layer as well as of carbon and surface groups. This was followed by a broad peak around 370 cm^−1^, showing the in-plane vibration of surface groups (T_x_) interacting with Ti atoms. Next, the peak located around 600 cm^−1^ was related to the vibrations of the carbon (both E_g_ and A_1g_ modes). Then, the weak peak at about 710 cm^−1^ may be related to H_2_O, because this peak was only observed in Ti_3_C_2_T_x_ dispersed in water in previous reports [44]. The common peaks in the above regions were observed for the samples as shown in Figure 4a. Moreover, the Raman spectra of Ti_3_C_2_T_x_ QDs with obvious fluorescence effect are shown in Figure S4b. It is noteworthy that the background fluorescence enhancement confirmed the fluorescence characteristics of the Ti_3_C_2_T_x_ QDs.

According to the XRD patterns, the diffraction peaks of the precursor Ti_3_AlC_2_ were exactly the same as those of the standard card of crystalline Ti_3_AlC_2_ (PDF #52−0875), as seen in Figure 4b. After etching, the broadened signal at ~6.3° was observed, which was the typical diffraction peak for the (002) plane of delaminated Ti_3_C_2_T_x_ MXene. The diffraction peak moved to a smaller angle compared to the 2*θ* value of 9.7° for the (002) plane of MAX. It was consistent with the increase of layer spacing after intercalation. On the other hand, the obvious peak at 39° and other characteristic peaks of MAX were absent, indicating the precursor had been completely delaminated. A series of diffraction peaks of the (00l) planes (l followed by 2, 4, 6, 8, 10) were often observed for delaminated Ti_3_C_2_T_x_. As for the as-prepared Ti_3_C_2_T_x_ NSs in this work, the diffraction peaks of the (0010) plane were observed, and other peaks were hard to distinguish from the background noise (Figure 4b). There was only a diffraction signal at a smaller angle for Ti_3_C_2_T_x_ QDs, without any diffraction patterns of oxides, especially titanium dioxide (TiO_2_) [45,46], which is often found in Ti_3_C_2_T_x_ QDs (Figure 4b). Although the exact diffraction peak position was difficult to determine due to the effect of low-angle incidence, it was certain that the QDs was pure Ti_3_C_2_T_x_ phase with a larger crystalline interplanar spacing. The crucial issues to prepare low dimensional MXenes are preventing oxidation and ensuring crystal quality, due to the facts that MXenes are fairly easily oxidized at ambient atmosphere or in O_2_-containing aqueous solution, especially for QDs, and defects resulted from the aggressive etching conditions (like high HF concentration) lead to poor crystal quality. Commonly, there have been diffraction peaks of titanium dioxide and a very broad diffraction signal of MXene’s (002) plane. In contrast, the Ti_3_C_2_T_x_ prepared in this work had better crystal quality and without oxidation.

To investigate the absorption properties of the Ti_3_C_2_T_x_ QDs, Figure 4c displays the absorption spectrum, revealing a broad strong absorption band from the ultraviolet to the visible region and a relatively weak absorption in the infrared band. The optical band gap values (*E*_g_) can be derived from extrapolating the graph of (*α*h*ν*)^2^ vs. h*ν* (the insert in Figure 4c), where *α* is the absorption coefficient, h is Planck’s constant, and *ν* is the photon frequency. The *E*_g_ of the Ti_3_C_2_T_x_ QDs was extrapolated to be 3.52 eV. The two-dimensional initial bulk Ti_3_C_2_T_x_ MXene was reported as a metallic band, and the *E*_g_ was relatively small, about 0.1 eV [29]. Therefore, the bandgap of Ti_3_C_2_T_x_ can be tuned from 0.1 to 3.52 eV by controlling its diameter from 2D to 0D.

### 3.2. Nonlinear Absorption of Ti_3_C_2_T_x_ QDs Tapered Fiber SA Device

**Figure 5:**
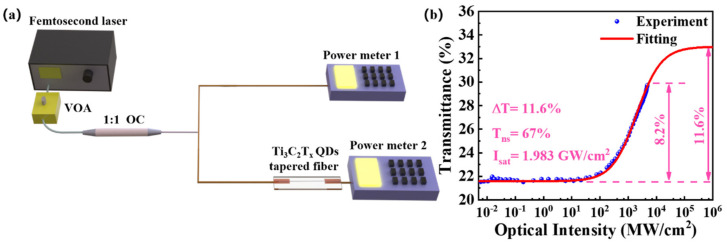
(**a**) Schematic diagram for the balanced twin-detector measurement system used for testing the Ti_3_C_2_T_x_ QDs tapered fiber SA device. (**b**) Nonlinear transmittance of the Ti_3_C_2_T_x_ QDs tapered fiber SA at wavelength of 1550 nm. It was fitted by a two-level saturable absorber model.

The nonlinear response of the Ti_3_C_2_T_x_ QDs tapered fiber SA was measured by a homemade balanced twin-detector measurement system as shown in Figure 5a. A femtosecond pulsed laser operated at a central wavelength of 1550 nm with a 151 fs pulse duration and a fundamental frequency of 47.6 MHz was divided into two beams by a 1:1 optical coupler (OC), and the power was recorded by two power meters. While power meter 1 was exploited for standard reference, power meter 2 was used to record the real-time power passing through the SA. The tunable incident power could be adjusted by a variable optical attenuator (VOA) to realize various transmittance values.

The experimental data of nonlinear saturable absorption at 1550 nm are plotted in Figure 5b, which were fitted by applying the well-known standard two-level saturable absorber model:

(1) $$T\left(I\right)=1-\frac{\Delta T}{1+\frac{I}{I_{sat}}}+T_{ns}$$

where *T*(*I*) is the transmittance, *I* is the incident optical intensity; the modulation depth (Δ*T*), saturation intensity (*I_sat_*), and nonsaturable absorption loss (*T_ns_*) were estimated to be around 11.6%, 1.983 GW/cm^−2^, and 67%, respectively. It is worth noting that the actual modulation depth (8.2%) did not reach the fitted value because of the large nonsaturation of the SA device, and the power of the test source also reached the upper limit. It may also have been caused by the high concentrations of quantum dots, which, similar to a previous report [47], possessed a larger nonsaturation absorption loss and modulation depth (Δ*T*). Moreover, the roughness, crystal defects, as well as free carriers of the material aggravated the light scattering and thus closely affected its related *T_ns_*. Compared to other high-dimensional SAs, QDs have more pronounced confinement and edge effects [48,49].Therefore, this result was supposed to contribute to the stronger nonlinear interaction of ultrasmall QDs applied in the tapered region.

### 3.3. Ultrafast Laser Application

**Figure 6:**
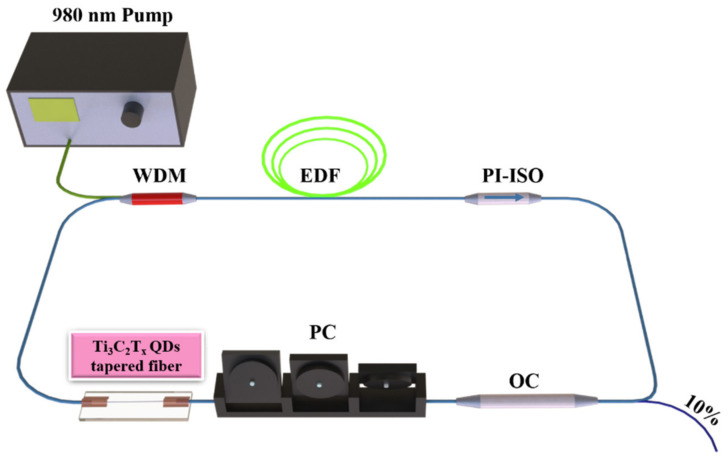
Structure diagram of the Er^3+^-doped fiber laser system.

The EDFL cavity based on the Ti_3_C_2_T_x_ QDs tapered fiber SA was set up to further verify the saturable absorption properties for pulse generation, which is schematically depicted in Figure 6. The ring cavity was pumped by a 980 nm single-mode diode laser (VLSS-980, Connet, Shanghai, China) with a 980/1550 nm wavelength division multiplexer (WDM). A 1.2 m Er^3+^-doped fiber (EDF, Er30, LIEKKI, Lohja, Finland) was used as the gain medium. A polarization independent isolator (PI-ISO, Thorlabs, Newton, NJ, USA) was employed to ensure the unidirectional operation of the laser in the ring cavity. The cavity polarization state and intracavity birefringence were adjusted by a polarization controller (PC, Thorlabs, Newton, NJ, USA). The SA based on Ti_3_C_2_T_x_ QDs was incorporated between the WDM and PC. Then, 10% of the laser separated by the OC (9:1) was used for detection, and the remaining 90% returned to the ring cavity to maintain resonance. In addition, a certain length of single-mode fiber (SMF) was needed for the ring cavity to output a signal. The output laser characteristics were monitored by an oscilloscope (MDO4102B, Tektronix, Beaverton, OR, USA). The output laser spectrum was detected by an optical analyzer (ASP-IR-2.6, Avesta, Moscow, Russia). The width of the mode-locked pulse was detected by an autocorrelator (FR-103XL, Femtochrome, Berkeley, CA, USA).

The group velocity dispersion (GVD) of EDF and SMF were 21.7 ps^2^/km and −27.6 ps^2^/km at 1550 nm, respectively. The cavity length was approximately 9.15 m. Therefore, the net dispersion could be calculated to be −0.1934 ps^2^. It means the EDFL operated in an anomalous region.

To eliminate the possible self-locking phenomenon of the laser in the experiment, we continuously adjusted the pump source power from 0 to 400 mW without inserting SA and slowly adjusted the PC at the same time. The laser was found to be operating in a continuous wave (CW) state with no indication of mode-locked or Q-switched phenomenon. After that, the SA was inserted into the laser cavity. Then, a self-started mode-locked pulse was observed when the pump power increased to 70 mW, and no polarization adjustment was required.

**Figure 7:**
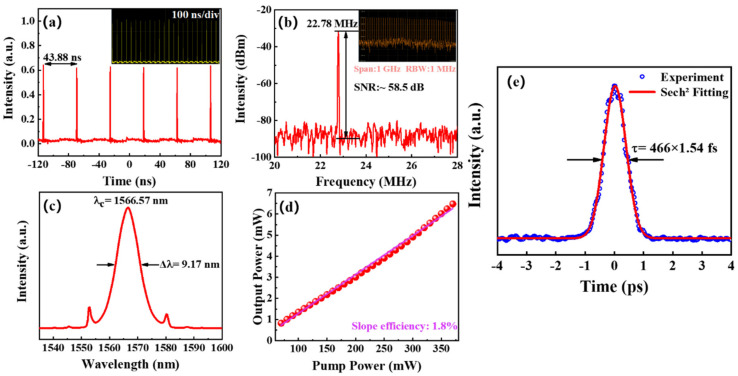
Ultrafast laser output characterizations of Er^3+^-doped fiber laser (EDFL) based on Ti_3_C_2_T_x_ QDs tapered fiber SA. (**a**) Mode-locking pulse trains. (**b**) The RF optical spectrum at the fundamental frequency. (**c**) Mode-locking optical spectrum. (**d**) The output power varies with pump power. (**e**) Autocorrelation trace with a sech^2^ fitting.

The characteristics of the EDFL output pulse are shown in Figure 7. When the pump power increased to 120 mW, the typical mode-locked pulse train was obtained as shown in Figure 7a. The uniform pulse train was acquired from the oscilloscope trace with a pulse interval of 43.88 ns, which precisely corresponds to the fundamental frequency of 22.78 MHz and the total cavity length of 9.15 m. Figure S5a (oscilloscope screenshot) shows a larger range of pulse trains within 1 μs, demonstrating the high stability of the pulses.

Figure 7b reveals a radiofrequency (RF) spectrum with a fundamental peak located at 22.78 MHz, which agrees well with previous results. The signal-to-noise ratio (SNR) was measured to be 58.5 dBm and a broadband RF spectrum within 1 GHz with a resolution bandwidth of 1 MHz was detected, as shown in Figure S5b (oscilloscope screenshot). It indicated that the laser was indeed working in a mode-locked state. The corresponding optical spectrum was centered at a 1566.57 nm wavelength with a full width at half-maximum (FWHM) of 9.17 nm as shown in Figure 7c. A pair of symmetrical Kelly sides on the spectrum can be clearly observed, suggesting the laser operated in the soliton pulse regime. The output power varied with the pump power, as illustrated in Figure 7d. The EDFL possessed a slope efficiency of 1.8% and it could achieve a mode-locked operation range from 70 to 370 mW, demonstrating a relatively low threshold value and the stability of laser operation over a wide range of power regulations. The maximum output power also reached 6.48 mW. Figure 7e presents the autocorrelation trace of the mode-locking pulse with a FWHM of 718 fs. Assuming it was a hyperbolic secant pulse, then the real pulse duration should have been 466 fs when the sech^2^ fit was used. Thus, the time-bandwidth product (TBP) could be calculated to be 0.552 by using the below equation:

(2) $$\mathrm{TBP}=\tau \times c\times \frac{\lambda_{\mathrm{FWHM}}}{\lambda_{c}^{2}}$$

where τ is the actual pulse width, *c* is the speed of light, λ_FWHM_ is the full width at half-maximum of the optical spectrum, and λ*_c_* is the central wavelength. The calculated value is slightly larger than the transform limit of 0.315 for the sech^2^ pulse, indicating that the pulse was chirped and there was the possibility to further reduce the dispersion of the cavity or broaden the spectral width to compress the pulse width. We summarized some of the reported mode-locking performances of quantum dot materials applied to EDFL, as listed in Table 1.

Compared with other research works on QDs materials, the laser performance of this work was relatively good and the as-prepared Ti_3_C_2_T_x_ QDs-based SA device was able to achieve femtosecond pulses with a higher modulation depth and lower saturation intensity. It is well known that a high modulation depth and low saturation intensity facilitate the mode selection of random longitudinal modes in the laser source by SAs. In turn, mode-locking is easily achieved to form a stable pulsed laser. Therefore, our SA device is more suitable for acquiring the mode-locked laser.

## 4. Conclusions

In summary, a MILD combined with an LPE method was employed to prepared ultra-thin Ti_3_C_2_T_x_ QDs (-F, -OH, =O, -Cl terminated) with an average lateral size around 5–10 nm, which was used to fabricate a tapered fiber SA device to apply in EDFL. Compared to other QDs SA devices, the as-prepared Ti_3_C_2_T_x_ QDs SA device possessed a higher modulation depth (11.6%) and lower saturation intensity (1.983 GW/cm^−2^). Mode-locked operation was successful realized via the SA device and showed a minimum pulse width of 466 fs with a low threshold power of 70 mW and a high output power of 6.48 mW. To the best of our knowledge, there are no reports of Ti_3_C_2_T_x_ QDs (-F, -OH, =O, -Cl terminated) serving as SAs at the communication band (1550 nm) by using the tapered fiber form to generate ultrafast lasers. This work is the first exploration of the application of this aforementioned form in ultrafast photonics. Meanwhile, considering the above excellent characteristics of the Ti_3_C_2_T_x_ QDs SA device, it will open up a new avenue for applications in ultrafast photonics.

## Figures and Tables

**Table 1:** Comparison of mode-locking performance of fiber lasers based on various QDs materials.

Materials	Fiber Platform	Central Wavelength (nm)	Bandwidth (nm)	Repetition Rate (MHz)	Pulse Duration (ps)	SNR (dBm)	Modulation Depth (%)	Saturation Level	Ref.
β-PbO	Fiber ferrule	1062.12	2	4.37	303	56	18.54	379.38 GW/cm^2^	[30]
PbS	Fiber ferrule	1563	4.78	13.9	0.559	68	44.5	-	[31]
S	Side-polished fiber	1530.6	3.9	8.32	0.720	60	-	-	[50]
CsPbBr_3_	Fiber ferrule	1600	4.5	8.528	14.4	50	2.5	17.29 MW/cm^2^	[20]
BP	Fiber ferrule	1567.5	2.4	15.22	1.08	64.3	36	3.3 GW/cm^2^	[51]
NbSe_2_	D-shaped fiber	1556	2.45	7.7	0.756	50	3.72	3.155 GW/cm^2^	[52]
GaTe	Side-polished fiber	1530.90	18.1	8.79	0.115	43	1.27	3.1 GW/cm^2^	[37]
SnTe	Side-polished fiber	1562.05	4.23	12.41	0.691	52	2.2	1.67 GW/cm^2^	[53]
Ti_3_C_2_T_x_	Tapered fiber	1566.57	9.17	22.78	0.466	58.5	11.6	1.983 GW/cm^2^	This work

## Data Availability

Not applicable.

## Supplementary Materials

The following supporting information can be downloaded at: https://www.mdpi.com/article/10.3390/nano12122043/s1, Figure S1: Schematic diagram of the tapered fiber SA and its image of tapered area under optical microscope; Figure S2: Schematic diagram of the preparation of self-assembled films for SPM and Raman testing; Figure S3: (a)Ti_3_C_2_T_x_ QDs’ thickness histogram with the average thickness. (b) Diameter histogram with the average diameter; Figure S4: (a) Raman spectrum of Ti_3_AlC_2_ MAX [54]. (b) Larger-scale Raman spectrum of Ti_3_C_2_T_x_ QDs with obvious fluorescence effect [55]; Figure S5: (a) Mode-locking pulse trains within 1 μs. (b) RF spectrum within 1 GHz with a resolution bandwidth (RBW) of 1 MHz.
