# Supplementary material for: Ti3C2Tx MXene Quantum Dots with Surface-Terminated Groups (-F, -OH, =O, -Cl) for Ultrafast Photonics
Source: Nanomaterials (Basel). 2022 Jun 14;12(12):2043. doi: 10.3390/nano12122043 (PMC9229704; doi:10.3390/nano12122043)
Supplement: Supplementary file 1 [file nanomaterials-12-02043-s001.zip › nanomaterials-1740399-supplementary.pdf]

nanomaterials

MDPI

Supporting Information

$Ti_3C_2T_x$ MXene Quantum Dots with Surface-Terminated Groups (-F, -OH, =O, -Cl) for Ultrafast Photonics

Jianfeng Liu [1], Shanshan Chen [1,2,*], Junshan He [1], Runming Huang [1], Lili Tao [1,2], Yu Zhao [1,2] and Yibin Yang [1,2]

1 School of Materials and Energy, Guangdong University of Technology, Guangzhou 510006, China; 2112002059@mail2.gdut.edu.cn(J.L.); hjs3108007371@163.com(J.H.); 3120006078@mail2.gdut.edu.cn(R.H.); taoll@gdut.edu.cn (L.T.); zhaoyu@gdut.edu.cn (Y.Z.); yangyibin@gdut.edu.cn (Y.Y.)
2 Guangdong Provincial Key Laboratory of Information Photonics Technology, Guangdong University of Technology, Guangzhou 510006, China
* Correspondence: chensunny@gdut.edu.cn

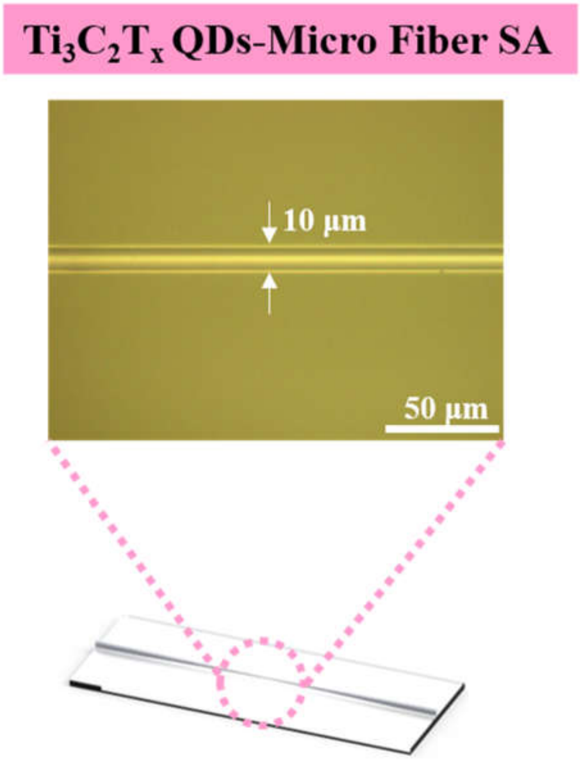

Figure S1. Schematic diagram of the tapered fiber SA and its image of tapered area under optical microscope.

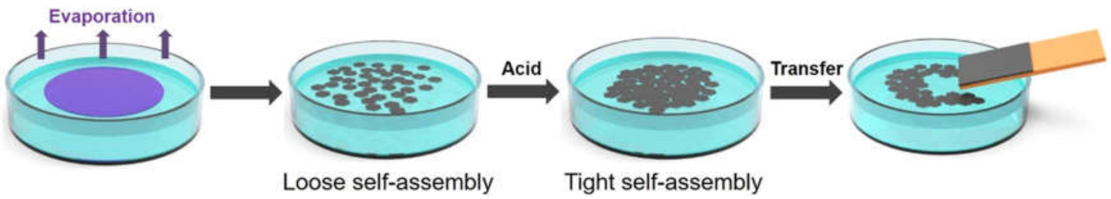

Figure S2. Schematic diagram of the preparation of self-assembled films for SPM and Raman testing. First, appropriate $Ti_3C_2T_x$-water colloidal solution was poured into a petri dish. Then, a small amount of ethyl acetate was added to the colloidal solution to make the $Ti_3C_2T_x$ self-assemble, thereby forming a $Ti_3C_2T_x$ film on the surface of the colloidal solution. Next, followed by the addition of a trace amount of 0.1 M HCl to the solution to make the $Ti_3C_2T_x$ film more dense. Finally, the self-assembled film was picked up using the substrates and full dried under vacuum before testing. Please refer to ref [1] for detailed process.

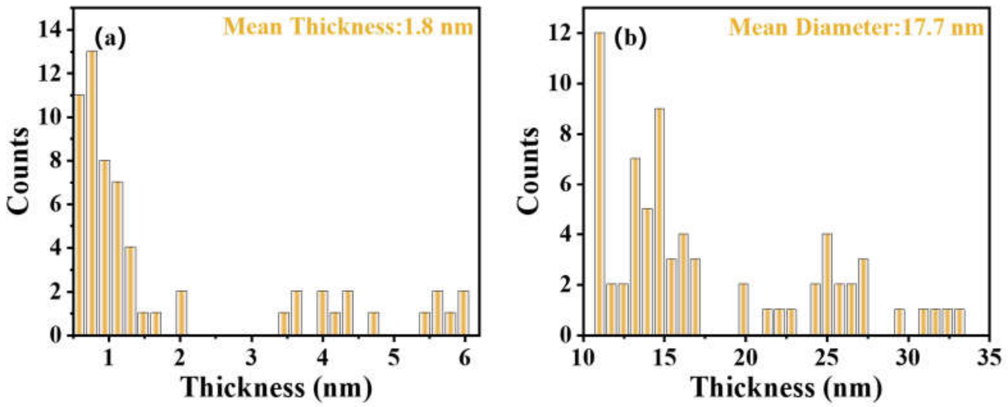

Figure S3. (a) $Ti_3C_2T_x$ QDs' thickness histogram with the average thickness. (b) Diameter histogram with the average diameter.

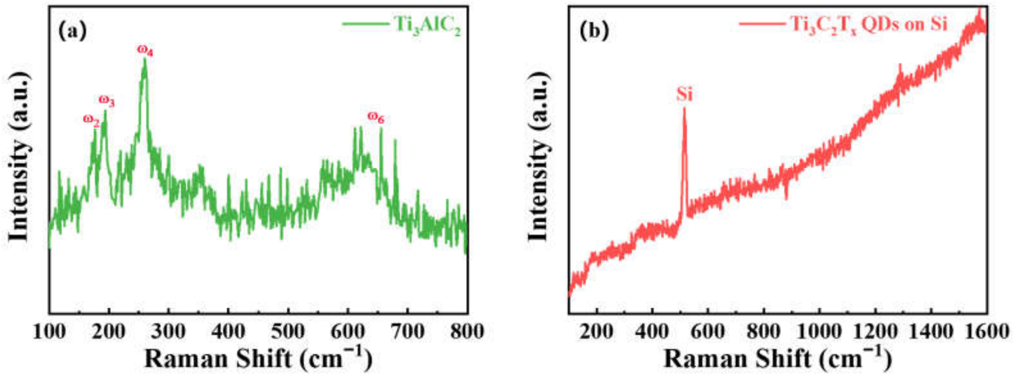

Figure S4. (a) Raman spectra of Ti_3AlC_2 MAX. (b) Larger scale Raman spectra of $Ti_3C_2T_x$ QDs with obvious fluorescence effect.

Consistent with the literature[2],ω2 (182 cm^{-1}), ω3 (199 cm^{-1}) and ω4 (270 cm^{-1}) belong to A_{1g} (Ti, Al) region, ω6 (660 cm^{-1}) belong to A_{1g} (C) in figure S4(a). Background fluorescence enhancement observed by larger scale Raman scanning of $Ti_3C_2T_x$ QDs as shown in figure S4(b), this phenomenon is also observed in lnP quantum dots[3], which appear more commonly in surface-enhanced Raman scattering (SERs) by using fluorescent molecules such as Rhodamine 6G (R6G).

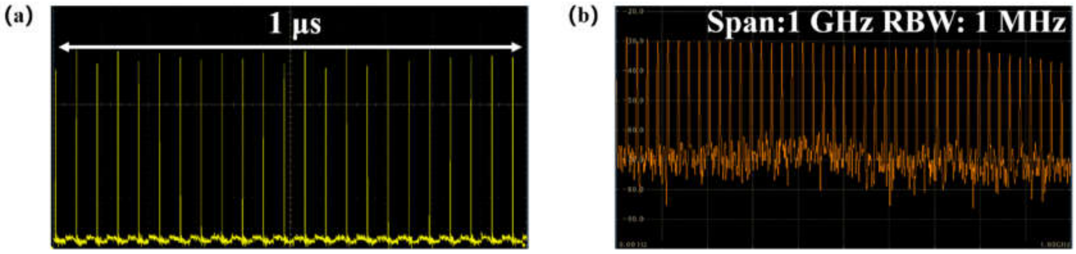

Figure S5. (a) mode-locking pulse trains within 1 μs. (b) RF spectrum within 1 GHz with a resolution bandwidth (RBW) of 1 MHz.

References

1. Kim, S.J.; Choi, J.; Maleski, K.; Hantanasirisakul, K.; Jung, H.-T.; Gogotsi, Y.; Ahn, C.W. Interfacial assembly of ultrathin, functional MXene films. *ACS Appl. Mater. Interfaces* **2019**, *11*, 32320-32327.
2. Sarycheva, A.; Gogotsi, Y. Raman spectroscopy analysis of the structure and surface chemistry of $Ti_3C_2T_x$ MXene. *Chem. Mater.* **2020**, *32*, 3480-3488.
3. Seong, M.; Mićić, O.I.; Nozik, A.; Mascarenhas, A.; Cheong, H.M. Size-dependent Raman study of InP quantum dots. *Appl. Phys. Lett.* **2003**, *82*, 185-187.
